# The Spectrum of Vestibular Disorders Presenting With Acute Continuous Vertigo

**DOI:** 10.3389/fnins.2022.933520

**Published:** 2022-07-13

**Authors:** Qingxiu Yao, Zhuangzhuang Li, Maoxiang Xu, Yumeng Jiang, Jingjing Wang, Hui Wang, Dongzhen Yu, Shankai Yin

**Affiliations:** ^1^Department of Otolaryngology-Head and Neck Surgery, Shanghai Jiao Tong University Affiliated Sixth People’s Hospital, Shanghai, China; ^2^Otolaryngology Institute of Shanghai Jiao Tong University, Shanghai, China; ^3^Shanghai Key Laboratory of Sleep Disordered Breathing, Shanghai, China; ^4^Department of Otolaryngology, The First Affiliated Hospital, College of Medicine, Zhejiang University, Hangzhou, China; ^5^Department of Otorhinolaryngology, ENT Institute, Affiliated Eye and ENT Hospital, Fudan University, Shanghai, China

**Keywords:** acute vestibular syndrome, vertigo, diagnosis, peripheral, central

## Abstract

**Objective:**

To explore the composition of vestibular disorders presenting with the acute vestibular syndrome (AVS).

**Methods:**

We performed a case analysis of 209 AVS patients between January 2016 and December 2020. These patients were grouped into different disorder categories according to the relevant diagnostic criteria.

**Results:**

We classified the 209 patients into 14 disorder categories, including 110 cases of vestibular neuritis, 30 of idiopathic sudden sensorineural hearing loss with vertigo, 17 of the first attack of continuous vertigo with migraine, 15 of Ramsay Hunt syndrome, 11 of acute labyrinthitis secondary to chronic otitis media, 8 of vestibular schwannoma, 6 of posterior circulation infarction and/or ischemia, 3 of cerebellar abscess secondary to chronic otitis media, 3 of AVS caused by trauma or surgery, 2 of AVS with down-beating nystagmus, 1 of multiple sclerosis of the medulla oblongata, 1 of epidermoid cyst of the posterior cranial fossa, 1 of a probable acute otolithic lesion, and 1 of AVS without measurable vestibular dysfunction.

**Conclusion:**

When a group of disorders present with AVS, characteristic clinical manifestations and imaging help with an accurate diagnosis.

## Introduction

Acute vestibular syndrome (AVS) is defined as the sudden onset of acute continuous vertigo associated with nausea, vomiting, motion intolerance, gait instability, and nystagmus lasting for days to weeks (Hotson and Baloh, 1998; Kattah et al., 2009; Chen et al., 2011; Newman-Toker et al., 2013a,b; Saber Tehrani et al., 2014; Mantokoudis et al., 2015b). A population-based descriptive study reported that 19.2% of dizzy patients have AVS, with an incidence of 92/100,000 population (Ljunggre et al., 2018). Although our understanding of AVS has improved recently, it is still not clear how many kinds of vestibular disorders present with AVS. Vestibular neuritis (VN) and stroke, which are both vestibular disorders that can provoke AVS, affect peripheral and central vestibular structures, respectively. However, other vestibular conditions, such as complications of chronic otitis media and demyelinating diseases (e.g., multiple sclerosis) are not well recognized, which can lead to delayed diagnosis, misdiagnosis, or improper treatment. Although progress has been made in basic and clinical vestibular studies, no histopathological, radiological, or physiological markers or other confirmatory diagnostic tests are available for diagnosing many disorders presenting as AVS; thus, such disorders are still diagnosed based only on clinical experience. Many conditions manifesting as AVS have not been well studied, such as “first attack of continuous vertigo with migraine.” In addition, due to the harm that AVS causes patients and the low efficiency of current diagnostic and treatment modalities, it is essential to improve our understanding of AVS.

Our previous study, with a relatively small sample size of 77 patients, classified AVS into various specific disorders (Yao et al., 2018a). The purpose of this study was to explore vestibular disorders that can be attributed to AVS. Therefore, we performed a case analysis of a relatively large number of AVS patients to explore the composition of vestibular disorders presenting with AVS. A definite or probable diagnosis was obtained for most patients based on the relevant diagnostic criteria.

## Materials and Methods

### Subjects

We retrospectively enrolled 209 patients with AVS who visited our outpatient clinic for vertigo and balance disorders at the Shanghai Jiao Tong University Affiliated Sixth People’s Hospital from June 2016 to December 2020. The study was performed in accordance with the recommendations of the Ethics Committee of the Shanghai Jiao Tong University Affiliated Sixth People’s Hospital [2020-KY-004 (K)].

### Inclusion and Exclusion Criteria

The inclusion criteria were acute-onset, persistent vertigo, dizziness, or instability lasting days to weeks (Venhovens et al., 2016). Exclusion criteria were a first attack duration of < 24 h, an episodic vestibular syndrome (EVS), such as Meniere’s disease or benign paroxysmal positional vertigo, and chronic vertigo or dizziness lasting longer than 3 months, such as bilateral vestibulopathy.

### Neuro-Otological Evaluation

In addition to symptom evaluation, the patients underwent examinations of the external auditory canal, tympanic membrane, and mastoid, as well as bedside examinations, and auxiliary examinations including pure tone audiometry (PTA), vestibular function testing [video head impulse test (vHIT), cervical vestibular-evoked myogenic potentials (cVEMPs) and ocular vestibular-evoked myogenic potentials (oVEMPs), and videonystagmography (VNG) test]. All patients took a horizontal head impulse test (h−HIT), 96.17% of patients took vHIT, 10.53% took cVEMPs and oVEMPs, and 33.01% took the VNG test. These tests were all performed in the acute stage.

Temporal bone computed tomography (CT) and craniocerebral magnetic resonance imaging (MRI) were performed where necessary. In our study, 21.53% of patients were screened using MRI, and all patients with abnormal ocular movements underwent MRI in the acute stage. Some patients with tinnitus and hearing loss, and whose symptoms did not improve after 3–7 days of treatment had MRI in remission stage.

### Disease Diagnosis

Most disorders were diagnosed according to the diagnostic criteria (Hunt, 1907; Vazquez et al., 2003; Zhang et al., 2007; Stachler et al., 2012; Jeong et al., 2013; Förster et al., 2016; Thompson et al., 2018; Greene and Al-Dhahir, 2020; Spinato et al., 2021). Idiopathic sudden sensorineural hearing loss (ISSNHL) with vertigo was defined as a subtype of sudden sensorineural hearing loss accompanied by acute continuous vertigo or imbalance, with an unknown etiology and cannot be attributable to another disorder. The probable acute isolated otolithic lesion was defined as those patients who presented with symptoms of AVS and had normal results of all the vestibular function tests except the VEMPs. AVS without measurable vestibular dysfunction referred to patients who had symptoms of vertigo or nausea, vomiting, motion intolerance, and gait instability, had normal results of vestibular function tests, and were not attributable to another disorder. Other disorders were diagnosed according to the specific clinical manifestations and auxiliary examinations, such as CT and MRI.

### Classification

#### Central and Peripheral Acute Vestibular Syndrome

Patients were grouped into different disorder categories according to the relevant diagnostic criteria. Vertigo caused by an impairment of the inner ear or vestibular nerve was classified as peripheral AVS. And the patients who had unilateral vestibular loss confirmed by vHIT and had normal MRI were classified into the group of peripheral AVS. If the lesion involved the vestibular nuclei, the vestibular connections of the brainstem, and/or the cerebellar circuits on CT or MRI, it was classified as central AVS (Bertholon et al., 2020).

### Statistical Analysis

All statistical analyses were performed with SPSS software (ver. 22.0; IBM Corp., Armonk, NY, United States). The positive rates of h-HIT and spontaneous nystagmus (SN) were compared between ISSNHL with vertigo and VN patients using the Chi-square test. *p* < 0.05 was considered to indicate statistical significance.

## Results

We enrolled 209 patients presenting with AVS. Based on the diagnostic criteria, 14 disorders were classified as peripheral or central AVS (Table 1).

**TABLE 1:** Comprehensive AVS classification system.

Classification	Case number	Male sex	Age (years)	Cardinal features (except vertigo)	Auxiliary examination	Peripheral/ Central AVS
Vestibular neuritis	110	56 (50.91)	46.35 ± 14.59	No hearing loss, no neurological symptoms or signs, no migraine	vHIT, VNG	Peripheral
Idiopathic sudden sensorineural hearing loss with vertigo	30	13 (43.33)	58.86 ± 11.13	Sudden hearing loss, no neurological symptoms or signs	PTA, vHIT, VEMP	Peripheral
First attack of continuous vertigo with migraine	17	3 (17.65)	42.29 ± 17.09	Migraine	vHIT, VNG, MRI	Peripheral/central
Ramsay Hunt syndrome	15	5 (33.33)	58.08 ± 15.45	Otalgia and auricular herpes, with or without facial nerve paralysis/hearing loss	vHIT	Peripheral
Acute labyrinthitis secondary to otitis media	11	2 (18.18)	61.91 ± 8.02	Otorrhea, hearing loss	vHIT, VNG, PTA, CT, MRI	Peripheral
Vestibular schwannoma	8	5 (62.50)	51.13 ± 12.10	Rapid hearing loss and/or tinnitus	vHIT, VNG, PTA, MRI	Peripheral
Posterior circulation infarction and/or ischemia	6	5 (83.33)	58.17 ± 10.02	Unstable gait, ataxia, motor and/or sensory aphasia, dysarthria, abnormal eye movements, gaze-evoked nystagmus	vHIT, VNG, MRI	Central
Cerebellar abscess or inflammation secondary to otitis media	3	2 (66.67)	61.33 ± 5.56	High fever, headache	vHIT, CT, MRI, VNG	Central
AVS caused by trauma or surgery	3	2 (66.67)	54.33 ± 4.19	Medical history of trauma or surgery	vHIT, CT, MRI, VNG	Peripheral/central
AVS with DBN	2	1 (50.00)	75.50 ± 1.50	DBN	vHIT, MRI, VNG	Peripheral/central
Multiple sclerosis of the medulla oblongata	1	0 (0.00)	19.00 ± 0.00	Instability, up-beating nystagmus	vHIT, MRI, VNG	Central
Epidermoid cyst of the posterior cranial fossa	1	0 (0.00)	43.00 ± 0.00	Instability, up-beating nystagmus	vHIT, MRI, VNG	Central
Probable acute otolithic lesion	1	0 (0.00)	52.00 ± 0.00	Only the VEMP is abnormal	vHIT, MRI, VNG, VEMP	Peripheral/central
AVS without measurable vestibular dysfunction	1	0 (0.00)	38.00 ± 0.00	Normal vestibular function	vHIT, MRI, VNG, VEMP	Peripheral/central

*AVS, acute vestibular syndrome; VEMP, vestibular-evoked myogenic potential; DBN, down-beating nystagmus; v-HIT, video head impulse test; VNG, videonystagmography.*

*Values of male sex represent numbers (percentages), and values of age represent mean ± SD.*

### Peripheral Acute Vestibular Syndrome

In total, 175 patients presented with peripheral AVS, including 110 cases of VN, 30 of ISSNHL with vertigo, 15 of Ramsay Hunt syndrome, 11 of acute labyrinthitis secondary to chronic otitis media, 8 of vestibular schwannoma, and 1 of AVS caused by trauma.

Of these patients, 145 patients had SN. The vHIT results showed that all the patients had unilateral vestibular dysfunction.

Vestibular neuritis is one of the most common peripheral causes of AVS, of which the diagnosis is generally based on a comprehensive interpretation of clinical and laboratory findings following reasonable exclusion of other disorders. Our study enrolled 110 patients with VN which accounted for 62.86% of peripheral AVS. No patient had hearing loss, and there were few patients with tinnitus and ear fullness, which were essential in differential diagnosis from ISSNHL with vertigo.

In our study, 30 patients were diagnosed with ISSNHL with vertigo. Thirty percent of patients were accompanied by tinnitus and 3.33% of patients were accompanied by ear fullness. Approximately 23.33% of patients were accompanied by nausea, vomiting, and gait instability. The positive rates of h-HIT (16.67% vs. 82.73%, *p* < 0.001) and SN (56.67% vs. 98.18%, *p* < 0.001) in ISSNHL with vertigo patients were significantly lower than those in VN patients.

Patients with Ramsay Hunt syndrome had unique symptoms and signs, including herpetic blisters on the skin of the external canal and auricle and severe otalgia (ear pain). In our study, 1 patient was accompanied with SN, and 1 patient with nausea and vomiting.

In total, 11 patients were diagnosed as acute labyrinthitis secondary to chronic otitis media. Patients performed history of chronic suppurative otitis media, ear pain, or discharge of pus from the external auditory canal. CT examination of the temporal bone was performed in all patients, 10 cases were accompanied by labyrinthine destruction, and there was no imaging manifestation of labyrinthitis in the other case, but only had symptoms of acute labyrinthitis.

Among the 8 patients with vestibular schwannoma, all patients had abnormal vHIT results, and most of them were subjected to lateral and superior semicircular canals.

### Central Acute Vestibular Syndrome

In total, 13 patients presented with central AVS, including one case of multiple sclerosis of the medulla oblongata, one of an epidermoid cyst of the posterior cranial fossa, three of cerebellar abscess or inflammation secondary to otitis media, six of posterior circulation infarction and/or ischemia, and two of AVS after intracranial surgery.

In these patients, two patients had up-beating nystagmus (UBN), two had down-beating nystagmus (DBN), two had gaze-evoked nystagmus, two had positional nystagmus, and four had horizontal SN.

**FIGURE 1:**
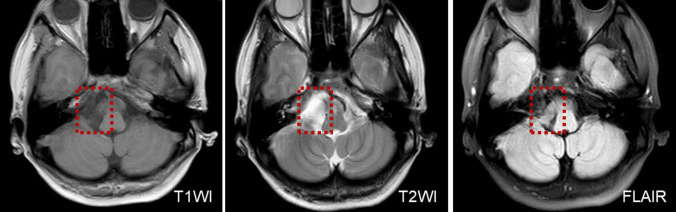
MRI shows low T1WI signal intensity and an inhomogeneous texture, in a patient with an epidermoid cyst of the posterior cranial fossa.

In two patients who presented with UBN, one patient was diagnosed with multiple sclerosis of the medulla oblongata. MRI was performed on suspicion of a central origin and revealed a demyelinating lesion in the medulla oblongata. The other patient was diagnosed with an epidermoid cyst of the posterior cranial fossa, this patient had a sudden onset of persistent vertigo with nausea and vomiting and perceived uneven ground while walking, perceived-up rotation of the surroundings when standing, and spontaneous UBN when the head was upright (Supplementary Video 1A) for the past month. The roll test revealed apogeotropic nystagmus on changes of direction (Supplementary Video 1B). MRI revealed abnormal signal intensities in the right cerebellopontine angle, manifesting as low signal intensity on T1-weighted images, and high signal intensity on T2-weighted and diffusion-weighted images (Figure 1).

**FIGURE 2:**
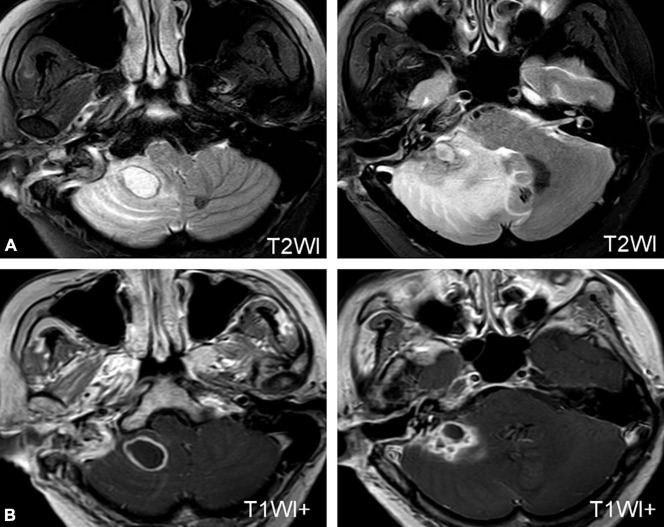
MRI of a patient with a cerebellar abscess. **(A)** MRI shows an abscess in the right cerebellum. **(B)** MRI of the patient after neurosurgery.

Three patients developed cerebellar abscesses or inflammation secondary to otitis media. One patient developed dizziness, headache, nausea, vomiting, and gait instability 5 days after a modified radical mastoidectomy. One patient developed severe vertigo, headache, and high fever while waiting for sinusitis surgery in the hospital. All patients were confirmed to have a cerebellar abscess or inflammation on MRI (Figure 2A) and were transferred to the Department of Neurosurgery for surgical treatment (Figure 2B).

**FIGURE 3:**
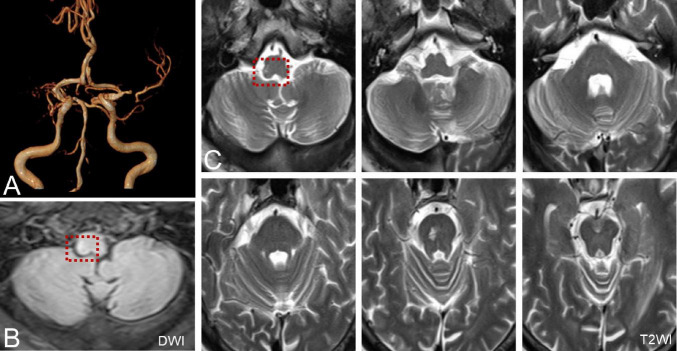
MRA and MRI of a patient with posterior circulation infarction. **(A)** MRA shows vertebrobasilar artery stenosis. **(B,C)** MRI shows acute infarction of the right medulla oblongata, which involved the ipsilateral vestibular nucleus (red dotted line frame).

**FIGURE 4:**
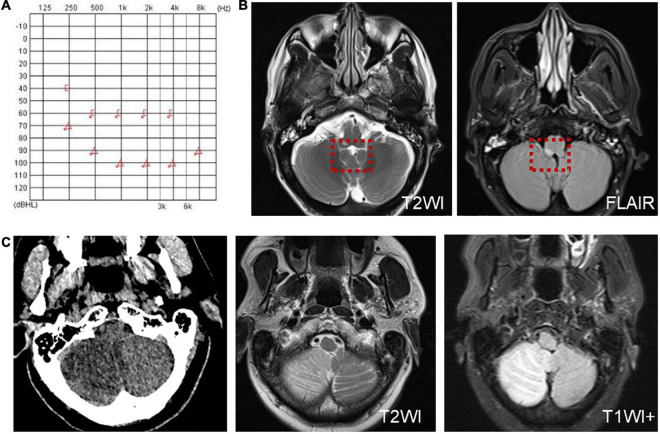
Examination results of patients with infarction of the cochlear and vestibular nuclei **(A,B)** and right cerebellar infarction **(C)**. **(A)** PTA shows total deafness on the right side in a patient with infarction of the cochlear and vestibular nuclei. **(B)** MRI shows that the infarcted focus of the right medulla oblongata near the pons involved the cochlear nucleus and vestibular nucleus (red dotted line frame). **(C)** Cranial CT and MRI of the patient with right cerebellar infarction.

Six patients were diagnosed with posterior circulation infarction and/or ischemia, including 1 case of focal infarction of the medulla oblongata, 1 of infarction of the cochlear, and vestibular nuclei, 2 of cerebellar infarction, and 1 of extensive infarction of the brainstem and cerebellum. One patient with a focal infarction of the medulla oblongata had SN (III°), obvious oculomotor abnormalities (including gaze-evoked nystagmus; Supplementary Video 2), and abnormal saccade and pursuit. The vHIT results showed reduced VOR gains of three semicircular canals on the right side, and magnetic resonance angiography (MRA) revealed vertebrobasilar artery stenosis (Figure 3A). MRI revealed acute infarction of the right medulla oblongata, which involved the ipsilateral vestibular nucleus (Figures 3B,C). The patient with infarction of the cochlear and vestibular nuclei presented with persistent dizziness, right facial discomfort, and decreased muscle strength in the right limbs. Backward tilting was seen during the Romberg test and the Fukuda test was unstable. PTA indicated total deafness on the right side (Figure 4A). MRI showed that the infarcted focus of the right medulla oblongata near the pons involved the cochlear nucleus and vestibular nucleus (Figure 4B).

Two patients were diagnosed with cerebellar lesions. One patient presented with vertigo, accompanied by gait instability, nausea, and vomiting. A physical examination and vestibular function test revealed SN to the right (II°); and positive Romberg and Fukuda tests. The caloric test and cVEMP indicated decreased vestibular function on the right side, whereas the vHIT was normal and accompanied by negative Babinski and Kernig’s signs. Cranial CT and MRI revealed a right cerebellar infarction (Figure 4C). The other patient presented with dizziness and sudden hearing loss on the right side, followed by the cerebellar herniation. Surgical treatment was applied in the Department of Neurosurgery.

**FIGURE 5:**
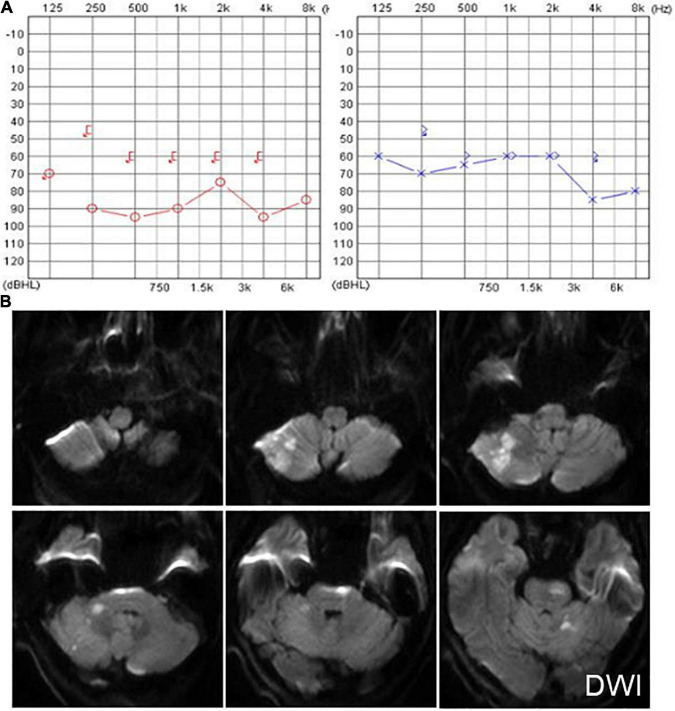
The PTA and MRI of the patient with extensive infarction of the brainstem and cerebellum. **(A)** PTA shows a bilateral sensorineural hearing loss in a patient with extensive infarction of the brainstem and cerebellum. **(B)** Brain MRI shows lacunar foci in the bilateral basal ganglia.

Another patient presented with continuous dizziness and sudden bilateral hearing loss; MRI revealed extensive infarction of the brainstem and cerebellum. PTA showed bilateral sensorineural hearing loss (Figure 5A), and brain MRI revealed lacunar foci in the bilateral basal ganglia (Figure 5B). The symptoms of both patients with cerebral infarction included vertigo with nausea and vomiting for 1 day, accompanied by DBN (Supplementary Video 3A). Brain MRI revealed bilateral basal ganglia and paraventricular infarcts. The other two patients had brainstem and basal ganglia ischemia and presented with DBN (Supplementary Video 3B).

**FIGURE 6:**
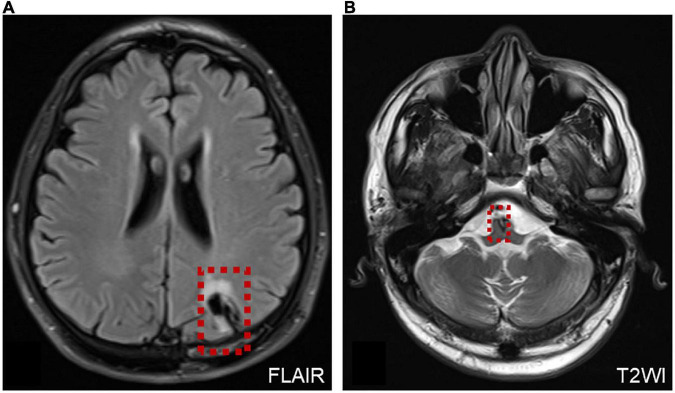
MRI of a patient with AVS after surgery. **(A)** MRI shows the operation site on the left side of the brain (red dotted line frame). **(B)** Malacia foci were detected in the left parietal lobe cortex and brainstem after craniotomy, and a few ischemic foci were scattered within the bilateral frontal–parietal lobe (red dotted line frame).

Two patients were diagnosed with AVS after surgery. One patient underwent microvascular decompression of the trigeminal nerve. The other patient presented with persistent oculomotor dysfunction after craniocerebral surgery, mainly characterized by horizontal–torsional nystagmus during the supine head-roll test (Supplementary Video 4). MRI revealed foci of malacia in the brainstem and left parietal lobe cortex after craniotomy (Figure 6).

### Indeterminate Diagnosis

In total, 17 patients presented with continuous vertigo, accompanied by migraine attacks during, before, or after the onset of acute vertigo. Among them, 16 patients with migraine presented SN and 1 patient had gazing-evoked nystagmus. In addition, 1 patient of AVS without measurable vestibular dysfunction, 2 patients with DBN and a normal MRI, and 1 patient with probable acute otolithic lesion who had abnormal cVEMPs and oVEMPs were also placed in this category.

## Discussion

Acute vestibular syndrome is characterized by sudden-onset acute continuous vertigo and has a variety of etiologies and manifestations. Both the peripheral and central vestibular systems may be involved. Accurate diagnosis is important for patients presenting with AVS, as it can manifest as a variety of conditions. In this study, we found that 209 patients can be classified as having 14 vestibular disorders, including both peripheral and central disorders.

Among these vestibular disorder categories, some of them are easy to diagnose, such as Ramsay Hunt syndrome. Ramsay Hunt syndrome is characterized by a vesicular rash on the outer ear with or without acute peripheral facial nerve paralysis (caused by reactivation of the varicella–zoster virus at the geniculate ganglion) (Haginomori et al., 2016). Ear pain and herpes zoster are characteristic symptoms and signs of Ramsay Hunt syndrome, which make it easy to differentiate this disorder from other types of AVS.

In our study, 17 patients had migraine attacks during, before, or after the onset of acute vertigo. This category included patients who had symptoms of migraine and vertigo at the first medical visit, therefore, they were diagnosed with the first attack of AVS with migraine. Vestibular migraine (VM) usually presents as EVS; however, it should be considered as migraine-related AVS when migraine is accompanied. Therefore, we classified patients who presented with their first attack of vertigo simultaneous with migraine into a separate category, which should be treated as migraine, not VN.

In most cases, ocular movements (gaze-evoked nystagmus, abnormal saccades and/or pursuit, UBN, and DBN) help differentiate central and peripheral vestibular lesions (Strupp et al., 2011), vHIT helps identify most peripheral AVS (Halmagyi et al., 2017; Yao et al., 2018b; Li et al., 2020), and MRI is a method to confirm central AVS. A recent study demonstrated that examining ocular movements could be better than MRI for diagnosing AVS with a central or peripheral etiology (Kattah et al., 2009). Oculomotor dysfunction and central forms of nystagmus are signs of central vestibular disorders. UBN is nearly always caused by central disorders; two of our patients presenting with UBN had lesions in or next to the medulla oblongata. However, patients with a repaired superior semicircular canal fissure may also have UBN (Sharon et al., 2017). DBN is also nearly always a clinical sign of a central nervous system abnormality, including structural lesions in the vestibulocerebellum, and of diseases such as multiple system atrophy, multiple sclerosis, cerebellar degeneration, and hydrocephalus (Yabe et al., 2003; Kattah and Gujrati, 2005; Lee et al., 2009). In our study, one patient with cerebral infarction and one patient with posterior circulation ischemia had DBN. Moreover, patients with central vestibular disorders may also exhibit abnormal pursuit and saccades, and gaze-evoked nystagmus. In our study, the patient with posterior circulation infarction had abnormal gaze-evoked nystagmus. Previous studies reported that nine in 30% patients with posterior circulation infarction showed abnormalities in the gazing test. Abnormalities in eye movement, especially gaze-evoked nystagmus and saccades, can help to screen posterior circulation infarction patients (Ling et al., 2019). In addition, one patient had persistent oculomotor dysfunction after craniocerebral surgery in our study. A previous study also reported that a patient showed new-onset UBN after microsurgical excision of the cavernoma (Meling et al., 2020), and two cases of purely UBN after bilateral superior canal plugging (Sharon et al., 2017). Therefore, abnormal ocular movements have a certain significance for the differential diagnosis of peripheral and central vestibular disorders.

Some of these disorders require a detailed differential diagnosis, including AVS with hearing loss and AVS with otitis media. AVS with hearing loss can include ISSNHL with vertigo, extensive infarction of brainstem and cerebellum, infarction of the cochlear and vestibular nuclei, and vestibular schwannoma. The patient developed a brainstem and cerebellar infarction with a fatal outcome. The diagnosis of ISSNHL with vertigo is based on symptoms, a physical examination, and auditory–vestibular function tests, rather than an etiological diagnosis. MRI is the modality of choice for diagnosing vestibular schwannoma, given its high sensitivity and specificity. Although vestibular schwannoma is a slow-growing tumor, some patients may also show acute vertigo attacks. Previous studies have shown that sharp changes in tumor size, such as tumor growth or intratumoral bleeding or cystic changes, can oppress the vestibular nerve or cerebellum, leading to acute vestibular symptoms. In addition, metabolic changes caused by tumor secretions may lead to sudden loss of vestibular function (Dilwali et al., 2015). Posterior circulation infarction can involve cochlear and vestibular nuclei, and/or the peripheral labyrinth, and presents as neurological symptoms and signs. MRI reveals the affected location. Notably, among posterior circulation infarctions, anterior inferior cerebellar artery territory infarction is most similar to ISSNHL with vertigo, as this artery mainly supplies peripheral vestibular and auditory structures (Lee, 2012; Kim and Lee, 2017). In addition, accumulating evidence indicates that vestibular symptoms could be secondary to otitis media, although AVS with otitis media has not been investigated (Monsanto et al., 2018). In total, 14 patients were diagnosed with AVS with otitis media. Patients in this category present not only with acute vertigo but also with typical symptoms of active otitis media, such as otalgia and otorrhea. Similar to sudden hearing loss, the complications of chronic otitis media can involve peripheral or central vestibular structures. In our study, 11 patients were diagnosed with acute suppurative labyrinthitis and three patients had intracranial complications. Foul-smelling purulent otorrhea, headache or severe pain in the deep ear, and fever are early signs and symptoms of intracranial complications caused by otitis media, neurological symptoms such as neck stiffness, and changes in mental status and gait ataxia are late manifestations (Schwaber et al., 1989). In addition, central signs such as oculomotor disturbances, directional-altering nystagmus, DBN, or UBN may be present. In patients with suppurative labyrinthitis, vHIT results showed abnormal gain and saccades. CT and MRI are of the highest importance to detect pathologies in suppurative labyrinthitis and intracranial complications (Kempf et al., 1998). Therefore, more attention should be paid to differentiating between peripheral and central involvement in AVS patients with hearing loss or otitis media.

In this study, patients attacked by acute vertigo after trauma or surgery were classified into a separate category. The vestibular system can be affected by trauma and surgery (Ibrahim et al., 2017; Marcus et al., 2019; Calzolari et al., 2021). Marcus et al. (2019) reported that, in 20% of cases, traumatic brain injury results in acute unilateral peripheral vestibular loss, typically due to petrous temporal bone fracture. Patients presenting with AVS after acute trauma should be monitored closely as their condition may deteriorate due to simultaneous injury of peripheral and central vestibular structures. The probable acute isolated otolithic lesion was also classified into a category, these patients who suffered from otolithic diseases occasionally perceive the visual world as tilted, or have an erroneous sensation of linear motion, postural unsteadiness, and neurovegetative symptoms such as nausea and vomiting (Denise et al., 1996).

Some disorders associated with AVS, such as labyrinthine hemorrhage, cerebral hemorrhage, and certain immune-mediated disorders, have not been included in our study. Other AVS disorders include Creutzfeldt–Jakob disease (Mantokoudis et al., 2015a), immune-mediated disorders such as cerebellitis and thiamine deficiency (Choi et al., 2007), medullary cavernous (Lee and Kim, 2017), primary central nervous system lymphoma involving the dorsal medulla (Lee et al., 2018), and functional and “psychiatric AVS”(Brandt and Dieterich, 2017).

Pseudo-vestibular neuritis (PVN) and acute ischemic stroke with audiovestibular loss (AISwAVL) are two types of malignant AVS. The clinical manifestations of PVN and VN are relatively similar. To differentiate PVN and VN, the results of nystagmus examination, head-shaking test, Fukuda and Romberg tests, and HINTS are useful. In addition, attention should be paid to whether the patient has disturbance of consciousness, headache, diplopia, and facial nerve palsy, dysphagia, and other central pathological signs, combined with the characteristics of central nystagmus, positive signs of ataxia, ocular movement disorders, and abnormal gravity perception (Ganggang et al., 2019). The clinical presentations of AISwAVL are similar to ISSNHL with vertigo. A previous study suggested that the HINTS “plus” hearing battery can help differentiate these two diseases (Saber Tehrani et al., 2014). Another study proposed that a combination of the HINTS and head-shaking nystagmus (HSN) can be useful. When the HINTS test is negative, HSN in the direction opposite to the SN and perverted HSN suggest the possibility of AISwAVL (Huh et al., 2013).

The HINTS examination can identify central AVS from peripheral AVS, relatively benign, and more common diagnostic alternatives (Kattah et al., 2009). vHIT provides an objective measurement of the VOR and can improve the diagnostic accuracy of AVS. vHIT combined with HINTS is a reliable tool to exclude posterior circulation stroke (Thomas et al., 2022). STANDING algorithm (spontaneous nystagmus, direction, head-impulse test, and standing) can be used by emergency physicians to independently identify central vertigo in AVS (Nakatsuka and Molloy, 2022).

Previous studies have identified various disorders presenting with AVS, including VN, brainstem lesions, and cerebellar infarction (Venhovens et al., 2016; Yao et al., 2018a; Kerber, 2020). However, those studies mainly focused on distinguishing central lesions involving the brainstem or cerebellum from VN; little attention was paid to other causes of AVS, such as otitis media and vestibular schwannoma (Venhovens et al., 2016; Yao et al., 2018a; Kerber, 2020). In this study, we enrolled a large number of patients to understand the composition of AVS, which can help deepen our understanding of AVS, and quickly distinguish peripheral from central AVS. A better understanding of the composition of acute vestibular disorders is helpful to make the correct diagnosis and differential diagnosis quickly. For example, some patients who initially show otitis media and vertigo need to be considered that they may be accompanied by labyrinthitis and even intracranial complications, which need to be diagnosed in time and avoid missed diagnosis. Furthermore, a variety of otological and neurological diseases, including some fatal diseases, manifest as AVS before a clear diagnosis, which requires the attention of clinicians. Therefore, classifying multiple types of AVS may help fast diagnosis and improve treatment outcomes, particularly for patients who need early and continuous vestibular rehabilitation.

This study had several limitations. As it was a retrospective study, the number of patients in some categories was small, and not all disorders with AVS were included. Patients in the retrospective study were enrolled from an ENT clinic which may introduce the selection bias, so the composition of the vestibular disorders in this study was different from those enrolled from an emergency room or neurology, which could not be generalized to other clinical settings. However, this study also shows that central AVS can also be seen in ENT practices, and otolaryngologists should also be vigilant to avoid missed diagnoses. In addition, we could not confirm whether the first attack of continuous vertigo with migraine developed into VM due to a lack of long-term follow-up data. It is necessary to continue to collect patient follow-up data to expand the spectrum of vestibular disorders presenting with AVS.

## Conclusion

This study enrolled a large case series and discussed the composition of vestibular disorders presenting with AVS and classified them into 14 categories according to the relevant diagnostic criteria, which can help deepen the understanding of AVS. Different from the previous AVS studies focusing on the differential diagnosis of vestibular neuritis and stroke, this study has an important significance for clinicians to broaden their list of differential diagnoses and consider other possibilities except for VN and stroke.

## Data Availability Statement

The original contributions presented in this study are included in the article/Supplementary Material, further inquiries can be directed to the corresponding author.

## Ethics Statement

The study involving human participants was reviewed and approved by the Shanghai Jiao Tong University Affiliated Sixth People’s Hospital. Written informed consent from the participants’ legal guardian/next of kin was not required to participate in this study in accordance with the national legislation and the institutional requirements.

## Author Contributions

DY and SY designed and coordinated the study. QY, ZL, and MX analyzed the data and wrote the manuscript. QY, ZL, MX, YJ, JW, and HW performed the data collection. DY attested that all listed authors meet authorship criteria and that no others meeting the criteria have been omitted. All authors contributed to the article and approved the submitted version.

## Conflict of Interest

The authors declare that the research was conducted in the absence of any commercial or financial relationships that could be construed as a potential conflict of interest.

## Publisher’s Note

All claims expressed in this article are solely those of the authors and do not necessarily represent those of their affiliated organizations, or those of the publisher, the editors and the reviewers. Any product that may be evaluated in this article, or claim that may be made by its manufacturer, is not guaranteed or endorsed by the publisher.

## Supplementary Material

Supplementary Video 1 Abnormal nystagmus in a patient with an epidermoid cyst in the posterior cranial fossa. **(A)** Spontaneous up-beating nystagmus. **(B)** Roll test examination shows apogeotropic nystagmus on changes of direction. Right beating nystagmus occurred when the patient was in the left supine position and left beating nystagmus occurred in the right supine position.

Supplementary Video 2 Abnormal gaze-evoked nystagmus in a patient with posterior circulation infarction.

Supplementary Video 3 Down-beating nystagmus. **(A)** Down-beating nystagmus in a patient with cerebral infarction. **(B)** Down-beating nystagmus in a patient with posterior circulation ischemia.

Supplementary Video 4 Persistent oculomotor dysfunction in a patient with AVS after craniocerebral surgery, mainly characterized by horizontal–torsional nystagmus during the supine head-roll test.
